# Supplementary material for: Sequences in the cytoplasmic tail of SARS-CoV-2 Spike facilitate expression at the cell surface and syncytia formation
Source: Nat Commun. 2021 Sep 9;12:5333. doi: 10.1038/s41467-021-25589-1 (PMC8429659; doi:10.1038/s41467-021-25589-1)
Supplement: Supplementary file 1 — Supplementary Information [file 41467_2021_25589_MOESM1_ESM.pdf]

Supplementary Information

# Sequences in the cytoplasmic tail of SARS-CoV-2 Spike facilitate expression at the cell surface and syncytia formation

Jerome Cattin-Ortolá[1,2], Lawrence Welch[1,2], Sarah L. Maslen[1], Guido Papa[1], Leo C. James[1] and Sean Munro[1]*

1: MRC Laboratory of Molecular Biology
Francis Crick Avenue
Cambridge CB2 0QH
UK

2: Denotes equal contribution
* Corresponding author: Email: sean@mrc-lmb.cam.ac.uk

## Contents:

Supplementary Figures 1-4

Supplementary Data 1-3 are provided separately as .xlsx files

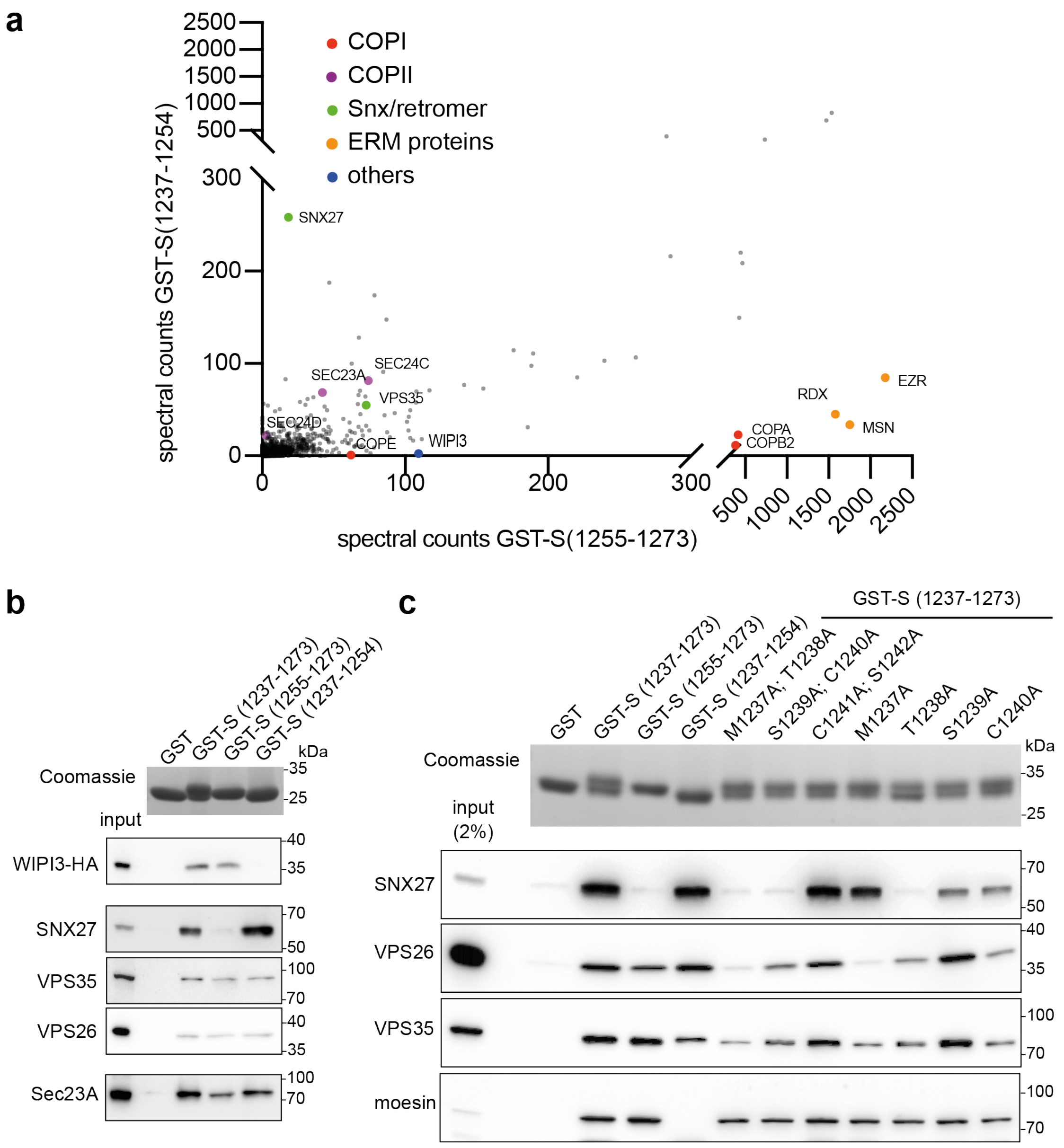


**Supplementary Fig. 1. Mapping of protein binding sites in the cytoplasmic tail of the SARS-CoV-2 S protein, including those of WIPI3 and SNX27/retromer**
**a** Plot comparing the spectral counts of proteins found by mass spectrometry analysis of interactors of membrane proximal GST-S(1237-1254) versus the membrane distal GST-S(1255-1273). The spectral counts are means from two biological repeats for GST-S(1237-1254) and three repeats for GST-S(1255-1273). All values in Supplementary Table 1.**b** The indicated GST-fusions expressed in *E. coli*, purified with glutathione Sepharose beads, and incubated with a 293T cell lysate followed by analysis of the bound proteins by immunoblot with the indicated antibodies. Coomassie-stained gel shows the GST-tail fusions. For WIPI3-HA, cells were transfected with a plasmid expressing the tagged protein and the blot probed for the HA tag. Representative blots from three independent experiments, input 1/50 of the lysate applied to the beads. **c** Mapping of residues required for binding to SNX27/retromer using the indicated GST-fusions as in (a) followed by probing with an anti-SNX27 antibody conjugated to HRP. Representative blots from three independent experiments, input 1/50 of lysate applied to beads.

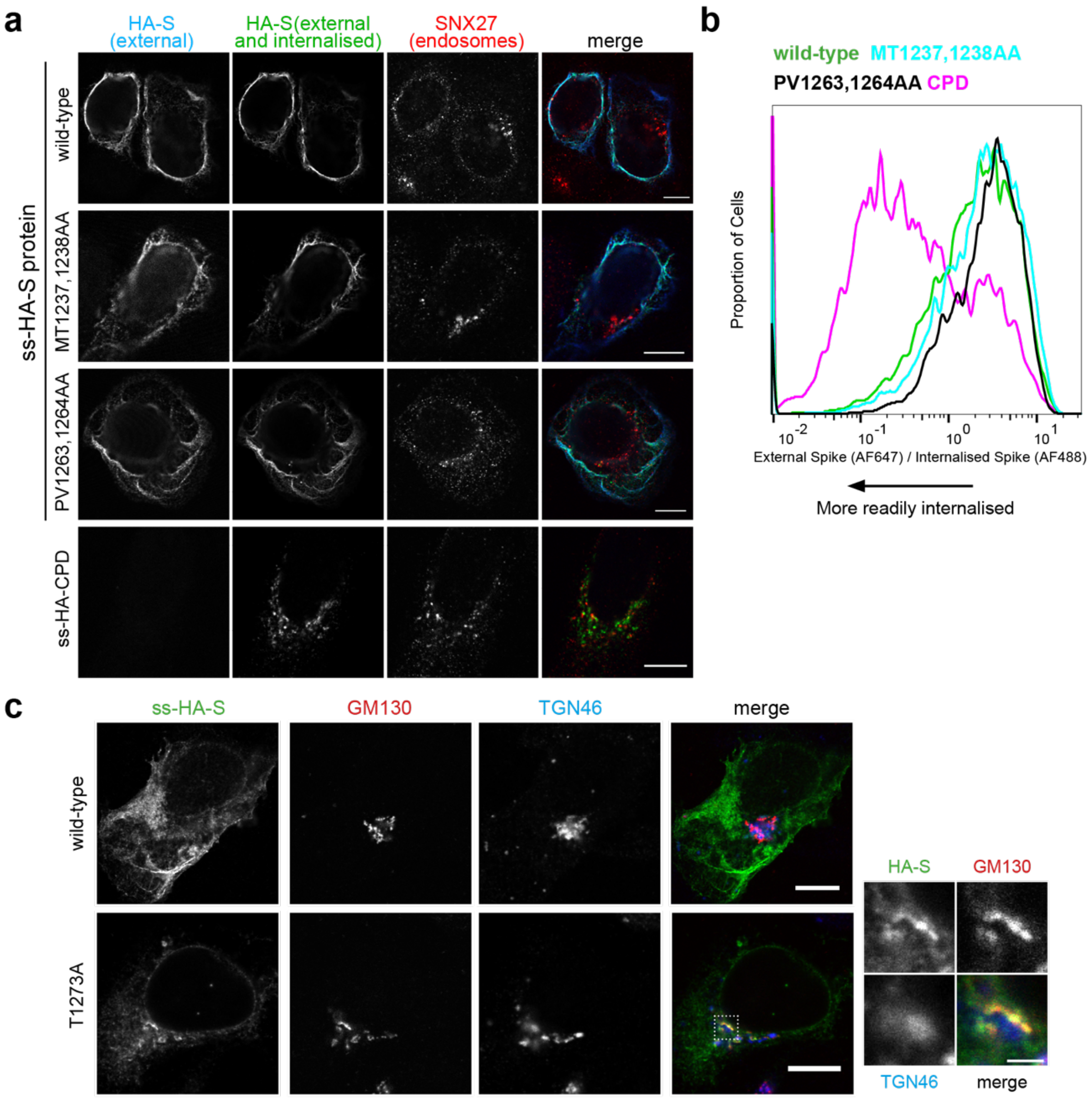


**Supplementary Fig. 2. The SARS-CoV-2 S protein is not rapidly internalised from the cell surface.**

**a** Micrographs of U2OS cells transiently expressing variants of S or carboxypeptidase D (CPD) (all with N-terminal HA tags), and incubated with an anti-HA antibody for 30 minutes on ice, unbound antibody washed off and cells chased for one hour in complete medium at 37ºC. Cells were fixed, and surface S labelled with AF647-conjugated secondary antibody under non-permeabilising conditions. After permeabilization, AF488-conjugated secondary antibody was used to label both surface and internalised S. Scale bars: 10 μm, with the images for the individual channels taken at the same magnification as the merged image. Representative of two biological replicates. **b** Flow cytometry to compare antibody uptake by HA-tagged S and CPD. Cells stained with AF488-conjugated anti-HA for 30 minutes on ice, washed, and chased for 40 minutes at 37ºC. Cells fixed and non-internalised anti-HA was simultaneously quenched and relabelled by incubation with an anti-AF488 antibody and an AF647-conjugated secondary antibody respectively under non-permeabilising conditions. Histograms represent the ratio of internalised S (AF488 signal) to that of non-internalised S (AF647 signal), normalised to the mode and representing 5000 - 10,000 events. Data representative of two independent biological replicates. **c** Confocal micrographs of U2OS cells transiently expressing N-terminally HA-tagged wild-type S or the T1273A variant, and labelled for GM130 (early-Golgi) and TGN46 (late-Golgi). Scale bars, 10 μm. Right panels show a zoom of the Golgi region within the white dotted rectangle, scale bar 2 μm. Images for the individual channels taken at the same magnification as the merged images with the scale bars. Representative of two biological replicates.

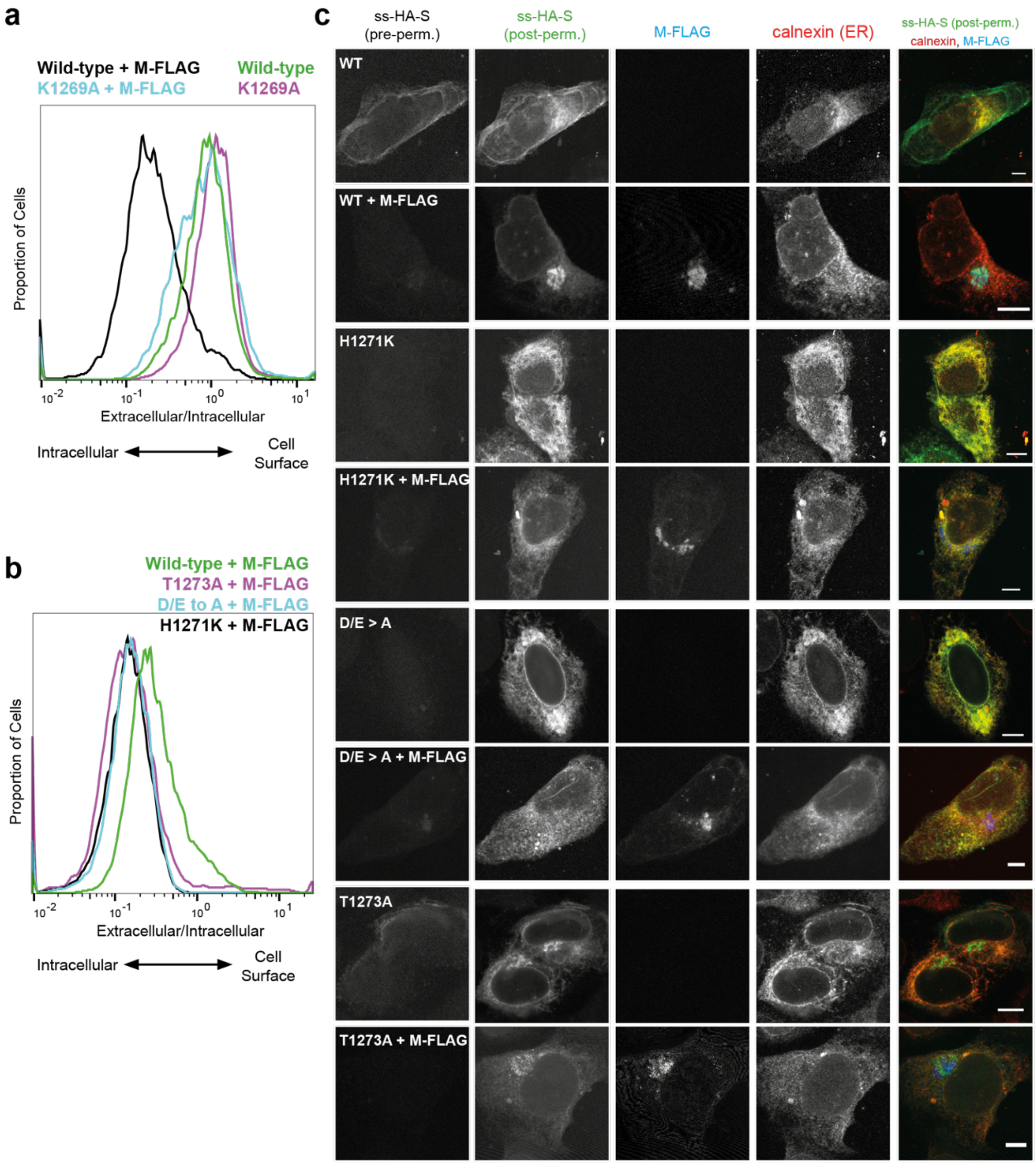


**Supplementary Fig. 3. Effect of co-expression of M protein on the localisation of S with mutations in the COPI and COPII binding motifs.**
**a** Histograms of the ratio of extracellular S (AF488) to intracellular S (AF647) for wild-type S and the K1269A mutant in the presence or absence of C-terminally FLAG-tagged M. As reported for SARS-CoV, Lys1269 of S is required for the protein to be retained in the Golgi by co-expressed M[1]. Histograms normalised to the mode of ~10,000 events. Representative of three independent repeats. **b** As (**a**), except that the ratio of the extracellular and intracellular levels of wild-type S in the presence of M is compared to those of the indicated variants of S in the presence of M. Mutations that reduce COPII binding, or increase COPI binding, reduce further the proportion of S that reaches the surface in the presence of M. Histograms normalised to the mode and represent ~10,000 events. Chi-squared tests show that in the presence of M, the differences between the median ratios of the wild-type (0.20) and of D/E>A (0.13), H1271K (0.11), and T1273A (0.12) are all statistically significant (P=0.01, 99% confidence). Representative of three independent repeats. **c** Confocal micrographs of U2OS cells transiently expressing N-terminally HA-tagged S or variants, in the presence or absence of M-FLAG. Unpermeablised cells (pre-perm.) were stained for the HA tag on S, and then cells permeabilised (post-perm.), and stained again for HA to label internal S, along with the FLAG tag on M, and the ER marker calnexin. Scale bars correspond to 10 μm, with the images for the individual channels taken at the same magnification as the merged images that have the scale bars. Representative of two biological replicates.

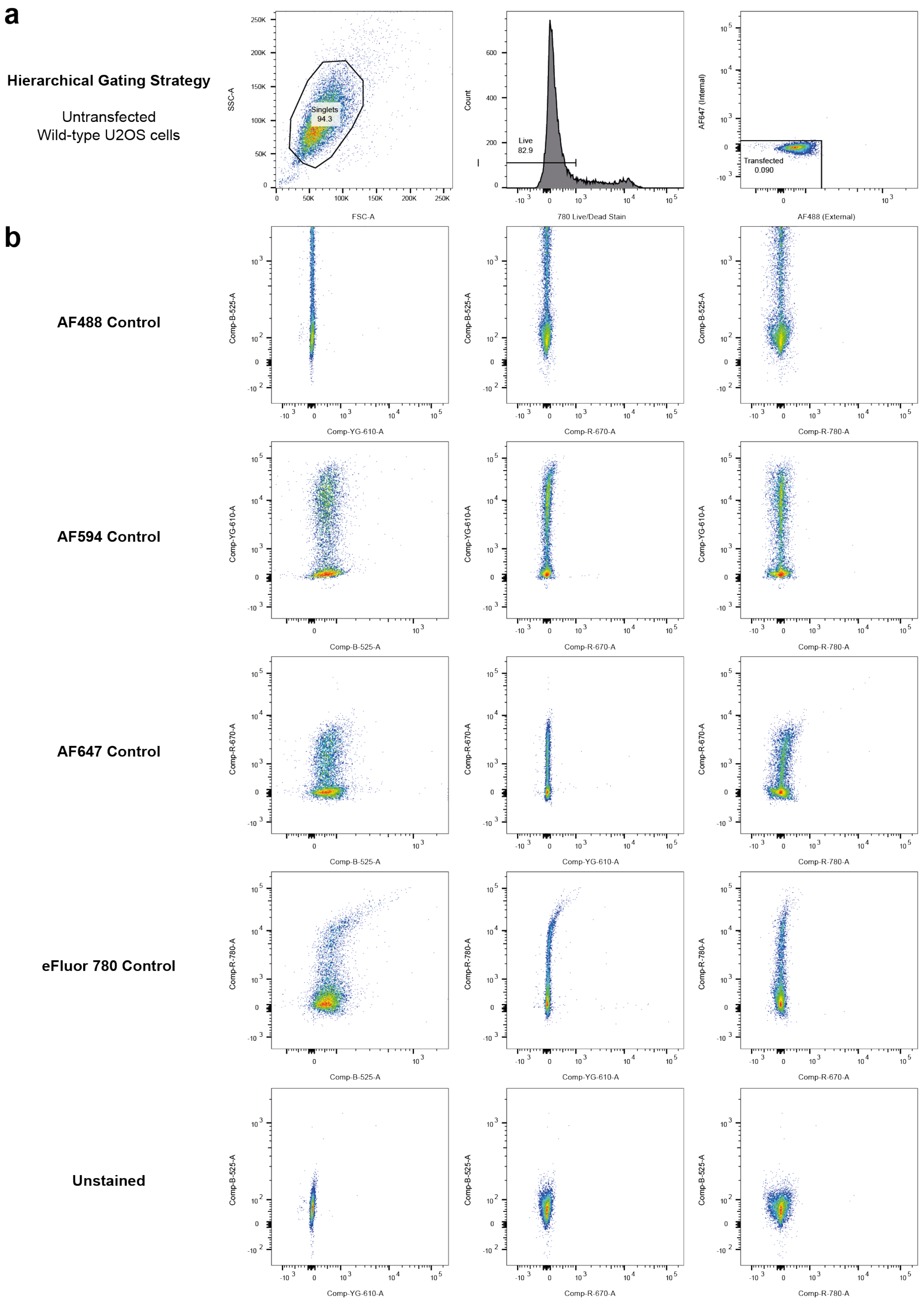


**Supplementary Fig. 4. Flow cytometry gating strategy and compensation controls.** **a** Wild-type, unstained U2OS cells were used to define the hierarchical strategy to gate for singlet, live, transfected cells. The hierarchy is displayed in order from left to right. Transfected cells were defined as cells with an Alexa Fluor AF488 or AF647 signal greater

than the unstained control. Forward scatter (FCS) and side scatter (SSC) are also shown. **b** Cells stained with a single antibody or dye were used as single colour compensation controls. Where required for single colour epitope stains, cells were transfected with a plasmid expressing an epitope-tagged protein. Comp-B-525-A (AF488), Comp-YG-610-A (AF594), Comp-R-670-A (AF647), Comp-R-780-A (eFluor 780).

**Reference**

1. McBride, C. E., Li, J. & Machamer, C. E. The cytoplasmic tail of the severe acute respiratory syndrome coronavirus spike protein contains a novel endoplasmic reticulum retrieval signal that binds COPI and promotes interaction with membrane protein. *J Virol* **81,** 2418–2428 (2007).
