# Supplementary material for: Sequences in the cytoplasmic tail of SARS-CoV-2 Spike facilitate expression at the cell surface and syncytia formation
Source: Nat Commun. 2021 Sep 9;12:5333. doi: 10.1038/s41467-021-25589-1 (PMC8429659; doi:10.1038/s41467-021-25589-1)
Supplement: Supplementary file 3 — Description of Additional Supplementary Files [file 41467_2021_25589_MOESM3_ESM.pdf]

Description of Additional Supplementary Files

File Name: Supplementary Data 1
Description: Summary of mass spectrometry data. Values are provided for spectral counts, and for spectral intensity with the latter analysed using a statistical test was a two-sided t-test, without adjustments for multiple comparisons.

File Name: Supplementary Data 2
Description: Plasmids, primers and antibodies.

File Name: Supplementary Data 3
Description: Flow cytometry statistical analysis. Values are provided for the median values, along with the chi-squared tests for significance.
