# Supplementary material for: Sequences in the cytoplasmic tail of SARS-CoV-2 Spike facilitate expression at the cell surface and syncytia formation
Source: Nat Commun. 2021 Sep 9;12:5333. doi: 10.1038/s41467-021-25589-1 (PMC8429659; doi:10.1038/s41467-021-25589-1)
Supplement: Supplementary file 7 — Reporting Summary [file 41467_2021_25589_MOESM7_ESM.pdf]

nature research

Corresponding author(s): Sean Munro
Last updated by author(s): Jul 16, 2021

Reporting Summary

Nature Research wishes to improve the reproducibility of the work that we publish. This form provides structure for consistency and transparency in reporting. For further information on Nature Research policies, see our Editorial Policies and the Editorial Policy Checklist.

Statistics

For all statistical analyses, confirm that the following items are present in the figure legend, table legend, main text, or Methods section.

n/a	Confirmed	
☐	☒	The exact sample size (*n*) for each experimental group/condition, given as a discrete number and unit of measurement
☐	☒	A statement on whether measurements were taken from distinct samples or whether the same sample was measured repeatedly
☐	☒	The statistical test(s) used AND whether they are one- or two-sided<br>*Only common tests should be described solely by name; describe more complex techniques in the Methods section.*
☒	☐	A description of all covariates tested
☒	☐	A description of any assumptions or corrections, such as tests of normality and adjustment for multiple comparisons
☐	☒	A full description of the statistical parameters including central tendency (e.g. means) or other basic estimates (e.g. regression coefficient) AND variation (e.g. standard deviation) or associated estimates of uncertainty (e.g. confidence intervals)
☐	☒	For null hypothesis testing, the test statistic (e.g. *F*, *t*, *r*) with confidence intervals, effect sizes, degrees of freedom and *P* value noted<br>*Give P values as exact values whenever suitable.*
☒	☐	For Bayesian analysis, information on the choice of priors and Markov chain Monte Carlo settings
☒	☐	For hierarchical and complex designs, identification of the appropriate level for tests and full reporting of outcomes
☒	☐	Estimates of effect sizes (e.g. Cohen's *d*, Pearson's *r*), indicating how they were calculated

Our web collection on statistics for biologists contains articles on many of the points above.

Software and code

Policy information about availability of computer code

Data collection	Mass-spectrometry data were collected with Proteome Discoverer v2.1 (Thermo Scientific). Confocal micrographs were collected with Leica Application Suite X. Cell fusions data were collected with IncuCyte software v2019B
Data analysis	Mass-spectrometry data was analysed using the Perseus module of MaxQuant v1.5.5.1. Flow cytometry data was analysed using FlowJo v10. Statistical analysis was with GraphPad Prism v8.

For manuscripts utilizing custom algorithms or software that are central to the research but not yet described in published literature, software must be made available to editors and reviewers. We strongly encourage code deposition in a community repository (e.g. GitHub). See the Nature Research guidelines for submitting code & software for further information.

Data

Policy information about availability of data

All manuscripts must include a data availability statement. This statement should provide the following information, where applicable:

- Accession codes, unique identifiers, or web links for publicly available datasets
- A list of figures that have associated raw data
- A description of any restrictions on data availability

Mass spectrometry data generated in this study have been deposited to the ProteomeXchange Consortium via the PRIDE partner repository with the dataset identifier PXD022215 [https://www.ebi.ac.uk/pride/archive/projects/PXD022215], and the processed data are summarised in Supplementary Table 1. All reagents generated by this study are available from the corresponding author on request.

Field-specific reporting

Please select the one below that is the best fit for your research. If you are not sure, read the appropriate sections before making your selection.

☒ Life sciences ☐ Behavioural & social sciences ☐ Ecological, evolutionary & environmental sciences

For a reference copy of the document with all sections, see nature.com/documents/nr-reporting-summary-flat.pdf

Life sciences study design

All studies must disclose on these points even when the disclosure is negative.

Sample size	Triple biological replicates for mass spectrometry were chosen based on accepted practice (Gordon et al (2020) Nature 583, 459-468). For flow cytometry, triple biological replicates of ~10,000 cells were used based on experience that this is sufficient to identify differences with a high degree of confidence.
Data exclusions	No data were excluded from the analysis, except for mass spectrometry where proteins whose identity had a low confidence were excluded based on a protein threshold set at 80%, a minimum number of peptides set as 2 and the peptide threshold set at 50%.
Replication	All experimental findings were reliably reproduced at least twice, and the number of such replicates is stated in the Figure Legends.
Randomization	Randomization was not applicable because we were comparing constructs where there was only a single difference between them. There were thus no possible confounding factors.
Blinding	For mass-spectrometry, flow cytometry, and cell fusion assays, blinding was not relevant as data collection was performed by machines and hence was free from observer bias, and the downstream analysis was with commercial software with quantitative outputs which were assessed by statistical methods, with all relevant data provided in the paper or in a repository. For experiments where proteins were separated on gels, or cells examined by microscopy, blinding was not felt necessary based upon experience and similar published studies. All experiments were repeated at least twice to ensure reproducibility, and typically examined by multiple authors. Key observations were independently validated using objective quantitative analysis, ie scanning gel lanes for gel based experiments, or flow cytometry for immunofluorescence.

Reporting for specific materials, systems and methods

We require information from authors about some types of materials, experimental systems and methods used in many studies. Here, indicate whether each material, system or method listed is relevant to your study. If you are not sure if a list item applies to your research, read the appropriate section before selecting a response.

Materials & experimental systems

n/a	Involved in the study	
☐	☒	Antibodies
☐	☒	Eukaryotic cell lines
☒	☐	Palaeontology and archaeology
☒	☐	Animals and other organisms
☒	☐	Human research participants
☒	☐	Clinical data
☒	☐	Dual use research of concern

Methods

n/a	Involved in the study	
☒	☐	ChIP-seq
☐	☒	Flow cytometry
☒	☐	MRI-based neuroimaging

Antibodies

Antibodies used	These are described in Supplementary Table 2.
Validation	Supplementary Table 2 provides for each antibody the Research Resource Identifier (RRID) from the Resource Identification Portal which provides information about validation and previous publications.

Eukaryotic cell lines

Policy information about cell lines

Cell line source(s)	293T, U2OS and Vero cell lines were from the American Type Culture Collection.
Authentication	Cell line authentification was not performed for the study.
Mycoplasma contamination	All lines tested negative for mycoplasma, using MycoAlert from Lonza.

Commonly misidentified lines (See ICLAC register)	No commonly misidentified lines were used.

Flow Cytometry

Plots

Confirm that:

- [x] The axis labels state the marker and fluorochrome used (e.g. CD4-FITC).
- [x] The axis scales are clearly visible. Include numbers along axes only for bottom left plot of group (a 'group' is an analysis of identical markers).
- [x] All plots are contour plots with outliers or pseudocolor plots.
- [x] A numerical value for number of cells or percentage (with statistics) is provided.

Methodology

Sample preparation	Antibody-labeled cells were resuspended in ice cold FACS buffer (2% FCS in PBS), and strained using a 100 µm filter prior to flow cytometry.
Instrument	An LSRII flow cytometer (BD Biosciences).
Software	FlowJo v10
Cell population abundance	No applicable as performing cytometry not cell sorting.
Gating strategy	Singlets were gated according to forward and side scatter profiles, dead cells were excluded using the viability dye and non-transfected cells were excluded based on their low AF488 and AF647 signals. Single colour controls were used to conduct compensation. This gating strategy is exemplified in Supplementary Fig. 4.

- [x] Tick this box to confirm that a figure exemplifying the gating strategy is provided in the Supplementary Information.
